# Functional properties of circular RNAs and research progress in gastric cancer

**DOI:** 10.3389/fonc.2022.954637

**Published:** 2022-11-16

**Authors:** Ping’an Ding, Pengpeng Liu, Haotian Wu, Peigang Yang, Yuan Tian, Qun Zhao

**Affiliations:** ^1^ The Third Department of Surgery, The Fourth Hospital of Hebei Medical University, Shijiazhuang, China; ^2^ Hebei Key Laboratory of Precision Diagnosis and Comprehensive Treatment of Gastric Cancer, Shijiazhuang, China

**Keywords:** Circular RNA, gastric cancer, marker, therapeutic target, molecular mechanisms

## Abstract

Circular RNAs (circRNAs) are a class of closed circular non-coding RNAs widely exist in eukaryotes, with high stability and species conservation. A large number of studies have shown that circRNAs are abnormally expressed in various tumor tissues, and are abundant in plasma with long half-life and high specificity, which may be served as potential tumor biomarkers for early diagnosis, treatment and prognosis of malignant tumors. However, the role of circRNAs is still poorly understood in gastric cancer. This article reviews the research progress of circRNAs in gastric cancer in recent years so as to explore the relationship between circRNAs and the occurrence and the development of gastric cancer, and provide new ideas for the diagnosis and treatment of gastric cancer.

## The formation and regulatory mechanism of circRNAs

### Formation of circRNA

CircRNAs are mainly divided into four categories according to their sources: exonic circRNAs (ecircRNAs) (1), intronic circRNAs (ciRNAs) (2), exon-intron circular RNAs (EIciRNA) (3) and intergenic circRNA (4). The biosynthesis of circRNA is different from the traditional canonical splicing mode of linear mRNA, but is formed by back splicing (5). Although the efficiency of circRNA reverse splicing is much lower than that of linear RNA, circRNA maintains high abundance in various species on accout of its high stability and long half-life. At present, the two formation mechanisms studied thoroughly are exon cyclization mechanism and intron cyclization mechanism.

Exonic circRNAs can be formed by reverse splicing of a single exon or multiple exons. The specific splicing methods are mainly divided into the following two models: the lasso-driven circularization model (6) and the intron pairing-driven circular model (7). The efficiency of the lasso-driven circularization model to form circRNAs is significantly higher than the intron pairing-driven circularization model, and it is a more common form of splicing in organisms (8).

Most exon-derived circRNAs are mainly formed through a lasso-driven circularization model. The mRNA precursor (pre-mRNA) is partially folded during transcription, bringing the distance between originally non-adjacent exons closer, resulting in exon skipping, thereby the crossed region forms the circRNA intermediates, and then forms the so-called exon circRNA through splicing.

During primary transcription, the intron regions on both sides of the primary mRNA(pri-mRNA) transcription product are complementary to each other due to the presence of reverse complementary sequences, such as ALU repeat elements, resulting in complementary pairing of introns and mediating the reverse splicing of exons and folding them together, and then partially cutting off introns to form some exonic circular RNAs.

The formation of intronic circRNAs is formed by the splicing of introns, and can be divided into group I introns, group II introns, and nuclear pre-mRNA introns (spliceosomal intron) according to different splicing methods. Most commonly, intron splicing during exonic circRNA formation is mediated by nuclear pre-mRNA introns, whereas intron circRNA formation is associated with class I introns and class II introns (9).

CircRNAs formed by intron cyclization can be produced in two ways: one is that an exogenous guanine nucleotide attacks the 5’ splice site, excises the 5’ exon and connects to the intron, and then the 3’ hydroxyl end of the excised 5’ exon attacks the 3’ splice site, releases the linear intron and joins the exons, and finally, the linear intron releases the 3’ tail to form a 2’-5’ junction intronic circular RNAs. The other is to first release the 3’ exon, and then the 2’ hydroxyl end of the intron attacks the 5’ splice site to generate circRNA.

**Figure 1:**
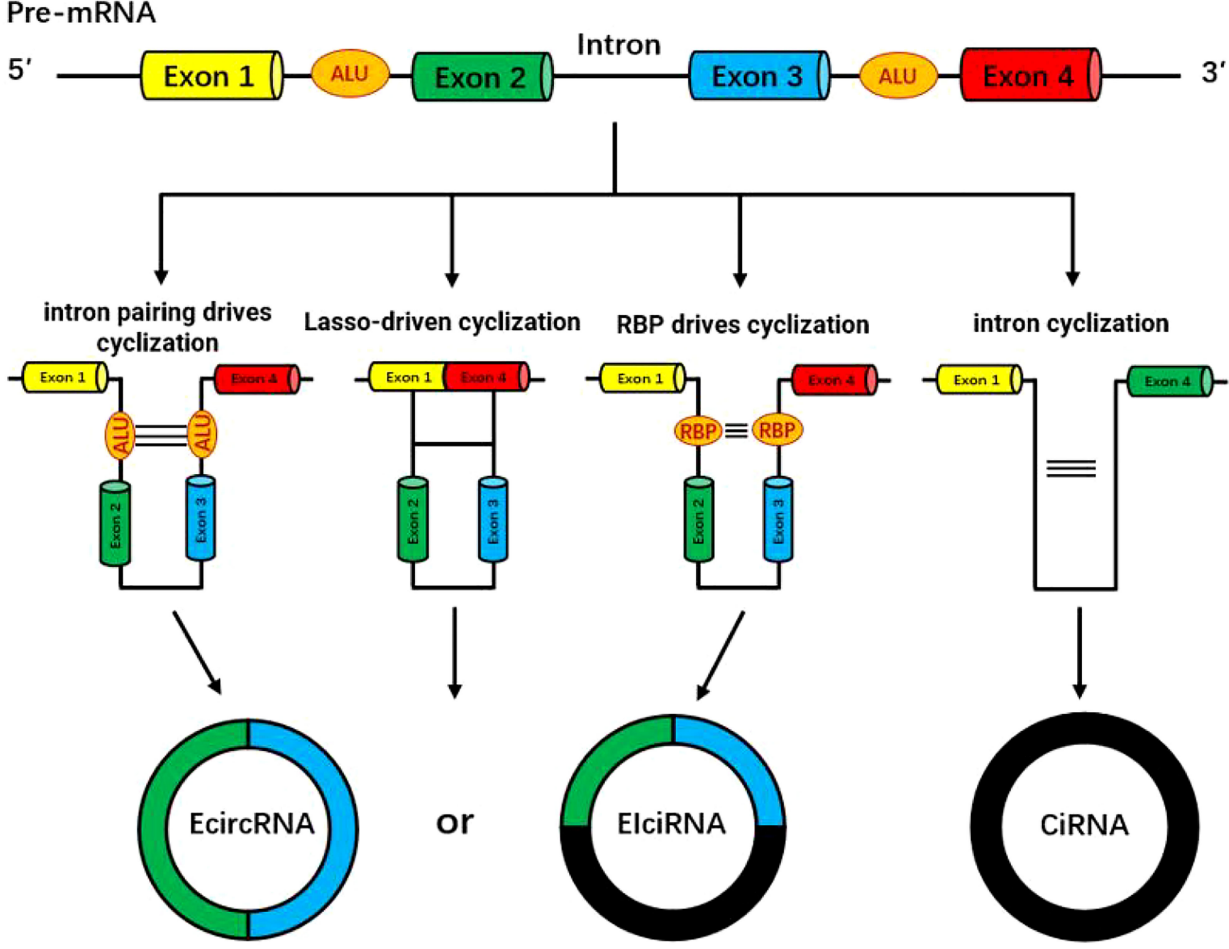
The formation and regulatory mechanism of circRNA.

An exogenous guanine nucleotide is used as a nucleophile to attack the 5 ‘ end splicing site, the exon at the 5’ end is excised by transesterification, and the exogenous guanine and intron are connected to each other, while the 3’ hydroxyl (-OH) above the cleaved 5’ terminal exon attacks the 3’ terminal splice site, resulting in the release of the linear intron by excision, the exons are connected to each other. Then the released linear intron removes its own 3’-terminal tail, and the remaining 2’-OH and 5’-terminal splice sites are connected to form a phosphodiester bond to generate an intronic circRNA (10, 11) **(** **Figure 1** **)**.

### The regulatory mechanism of circRNA

Mechanistic studies have shown that the flanking intron regions of circRNA circularized splice junctions usually contain reverse complementary sequences of varying lengths, including a repeat sequence of about 30-40 nucleotides that exists in primate genomes, called ALU element, and this structure can significantly promote the formation of circRNA (12). In addition to the above-mentioned Alu elements, it is reported that the complementary sequences within certain exons and their flanking introns can promote RNA reverse splicing to form circRNAs through base pairing (13).

The formation of circRNAs may also be affected by RNA-binding proteins (RBPs). On the one hand, RBPs can promote the formation of circRNAs by binding to target sites in the flanking intronic regions of pre-mRNA. For example, the splicing factor Quaking promotes circRNA formation by binding to targets upstream and downstream of circularized flanking intron regions on SMARCA5 pre-mRNA (14). The immune factors NF90 and/or NF110 promote circRNA production by binding to the inverted repeat Alus (IRAlus) of pre-mRNA (15). On the other hand, RBPs can also inhibit the formation of circRNAs by affecting the RNA pairing process. For example, the adenosine deaminase ADAR1 affects RNA pairing that A-to-I editing circularized exon flanks, reducing the complementarity and stability of the RNA pairing and inhibiting circRNA formation (16). The RNA helicase DHX9 can bind to IRAlus and inhibit circRNA formation by unraveling the paired RNAs flanking the circular exons (17).

In addition, circRNA formation occurs in synergistic transcription and post-transcriptional coupling with Pol II transcription (18, 19). A study on Pol II transcription elongation (TER) of genes that promote circRNA formation (20) found that the average TER of circRNA was higher than that of non-circRNA, and the change of TER had a significant effect on the formation of circRNA. They believed that this might because higher TER can allow the transcription of downstream intron complementary sequences (ICS), increasing the possibility of ICS cross-exon pairing, thereby increasing the possibility of reverse splicing circRNA formation. At the same time, higher TER-related linear splicing reduction may also promote the formation of circRNA. There are also quite a few circRNAs that are formed after transcription. A large number of nascent circRNAs are detected only after the transcription of their host pre-mRNAs has been completed (20). Further Mechanistic studies have shown that mRNA 3 ‘ -end processing is required for circRNA production (2), and inhibition of co-transcription 3 ‘ -end processing can increase circRNA formation (21), which can be attributed to increased transcription of Pol II upstream polyadenylation signals.

## Biological functions of circRNAs

### Interaction between circRNA and miRNA

**Figure 2:**
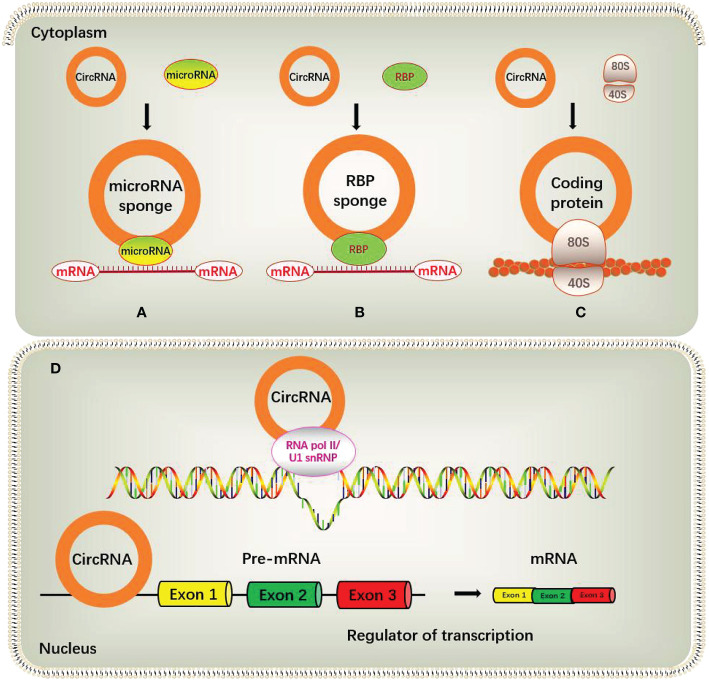
Biological functions of circRNAs.

Some circRNAs contain miRNA binding sites, and act as competing endogenous RNAs (ceRNAs) to negatively regulate miRNA activity by competing miRNA binding sites, thereby reducing the inhibitory effect of miRNAs on their downstream target genes. The most representative example is the antisense transcript of cerebellar degeneration-related protein-1 (CDR1as), also known as ciRS-7, which contains 74 selectively conserved miRNA-binding sites and acts as a molecular sponge for miR-7. Another circular RNA circSRY, exists specifically in mouse testis tissue, contains 16 binding sites for miR-138 (22, 23). Another study found that circHIPK3 could act as a molecular sponge for miR-124, upregulate the expression of miR-124 target genes IL6R and DXL2, and then promote the proliferation of liver cancer cells (24). But it is worth noting that the concentration of circRNA and corresponding miRNA should be at a similar level to facilitate effective competitive binding of miRNA, and only in this way, circRNAs can effectively bind miRNAs and inhibit their functions. However, many circRNAs in organisms are usually expressed in low abundance and lack multiple targets for the same miRNA molecule, so the function of miRNA sponge is limited (25) **(** **Figure 2A** **)**.

Besides acting as miRNA sponges and specific inhibitors of target miRNAs, circRNAs can also act as miRNA reservoirs to stabilize or activate miRNA functions. For example, ciRS-7 can be considered as miR-7 reservoir. After miR-671 cleaves ciRS-7, miR-7 is released in large quantities and its activity increases rapidly, consequently resulting in the effective inhibition of miR-7 targets. At this time, ciRS-7 becomes a “miR-7 reservoir” and activates miR-7 function (26). ERβ represses the circular RNA circATP2B1, which acts as a reservoir of miR-204-3p by transcription. The decreased circATP2B1 cannot stabilize the expression of miR-204-3p, resulting in the decrease of miR-204-3p, which in turn increases its downstream target FN1 and enhanced the invasive ability of ccRCC cells (27). Other studies have shown that circ-HIAT1 can act as a reservoir for miR-195-5p, miR-29a-3p and miR-29c-3p to stabilize the expression of these miRNAs, thereby inhibiting the activity of the downstream target gene CDC42 (28).

### circRNA interacts with RBP

RBP is a class of proteins widely involved in gene transcription and translation in organisms, and the interaction with RBP can be considered as an important part of the function of circRNA. RBPs function in splicing, processing, folding, stability and localization of circRNAs by interacting with the circularized splice junctions of circRNAs (29). For example, circ-Foxo3 can form a Foxo3-p21-CDK2 ternary complex by interacting with cyclin-dependent kinase 2 (CDK2) and p21, resulting in the inhibition of CDK2 function and the blockage of cell cycle progression, thereby regulating tumor development (30). In addition, circ-Foxo3 was also found to bind to two proteins, MDM2 and p53, promoting MDM2-induced p53 ubiquitination and subsequent degradation (31). Another study found that the circular RNA circPABPN1 inhibited the combine of HuR with linear PABPN1 mRNA by competitively interacting with HuR, thereby affecting the translation of its cognate transcript PABPN1 (32) **(** **Figure 2B** **)**.

### circRNAs can regulate gene transcription

Nuclear EIciRNAs and ciRNAs may be involved in the transcriptional regulation of genes. Studies shown that the transcription of the parental gene was inhibited after knockdown of EIciRNA. Mechanistic studies have shown that EIciRNAs such as circEIF3J and circPAIP2 form EIciRNA-U1 snRNP complexes by interacting with U1 small nuclear ribonucleoprotein (U1snRNP), and interacte with RNA polymerase II (Pol II) on the promoters of EIciRNA parent genes to promote transcription of the parental gene. In addition, when the above RNA-RNA interaction is blocked, the interaction between EIciRNA and Pol II is disrupted, resulting in a subsequent decrease in the transcription of the EIciRNA parental gene (3). Another study found that circSEP3, a circular RNA derived from exon 6 of SEPALLATA3 (SEP3) in Arabidopsis thaliana, could tightly bind to its cognate DNA locus to form a circRNA : DNA complex, which could cause transcriptional pause, and exon-skipping alternatively spliced SEP3 mRNA (33). Besides, abundant ciRNAs such as ci-ankrd52 in the nucleus may positively regulate Pol II transcription by extending the Pol II mechanism. When ci-ankrd52 is knocked down, the transcription of its parental gene is also reduced (2) **(** **Figure 2D** **)**.

### circRNAs can encode polypeptides

Although circRNAs have always been defined as non-coding RNAs, some circRNAs have internal ribosome entry site elements (IRES) (34) or prokaryotic ribosome binding sites (35), so these circRNAs are no longer non-coding RNA in the traditional sense, but a special circular RNA capable of encoding polypeptides. Given circRNAs lack caps and poly(A) tails, thus, translation of circRNAs may occur in a cap-independent manner. At present, with the continuous development of circRNA research, many online databases can be referenced for researchers. Such as, the circRNADb database contains the specific information of 32,914 human exonic circRNAs, including the genome sequence, open reading frame (ORF) and IRES. This information can help us to assess whether circRNAs have coding potential (36). For example, circZNF609 can encode functional polypeptides and participate in regulating the proliferation of myoblasts during muscle differentiation (37). Another circular RNA, circMbl, derived from the muscleblind (Mbl) locus, encodes a protein that has been detected in fly head extracts by mass spectrometry (38).

### Interaction between circRNA and mRNA

Mechanistic studies have shown that CircRNA can not only bind to mRNA, but also act as a regulator of mRNA translation and stability (39, 40). For example, CircIPO11, a regulator necessary for liver cancer stem cells (CSCs) to maintain self-renewal, can recruit the TOP1 to the GLI1 promoter to trigger its transcription, thereby activating the Hedgehog signaling to promote liver CSC self-renewal and HCC progression (40). circYAP is a circRNA that regulates the translation of mRNA. In the translation initiation complex, circYAP can specifically recognize and bind to YAP mRNA, eliminating the interaction between PABP on the poly (A) tail and eIF4G on the 5 ‘ -cap of Yap mRNA, resulting in the inhibition of Yap translation initiation (41). CircRNA can regulate mRNA stability. When circXPO1 binds to IGF2BP1 and enhances the stability of CTNNB1 mRNA, the inhibitory effect of CTNNB1 is enhanced, thereby accelerating the progression of lung tumors (42). Similarly, circARHGAP12 enhances the stability of EZR mRNA by binding to the 3’UTR of EZR mRNA, thereby promoting the progression of nasopharyngeal carcinoma (NPC) (39, 43).

In addition to the above mechanisms, circRNAs may also initiate the translation process through N6-methyladenosine (m6A) modification. CircRNAs are rich in m6A consensus motifs, and a single m6A site is sufficient to drive translation initiation. One study found that m6A-driven circRNA translation was widespread through ribosome sequencing analysis and mass spectrometry detection, and many endogenous circRNAs had translation potential (44) **(** **Figure 2C** **)**.

## The relationship between circRNA and gastric cancer

With the deepening of circRNA research in recent years, the relationship between circRNA and various human diseases has been discovered one after another, including difficult-to-treat tumors. Gastric Cancer (GC) is one of the most common malignant tumors of the digestive system in the world, with higher morbidity and mortality in our country (45). Although great progress has been made in the treatment of gastric cancer, the five-year overall survival rate of patients with gastric cancer is still low due to the high clinical heterogeneity and the variable progression of gastric cancer (46). Therefore, there is an urgent need to find effective biomarkers for early diagnosis, early treatment and prognostic of gastric cancer, so as to provide more timely and precise treatment options for gastric cancer patients. Recently, some studies have emerged on the abnormal expression and mechanism of circRNAs in gastric cancer, which may have important implications for the diagnosis, treatment and prognosis of gastric cancer.

### Differential expressions of circRNAs in gastric cancer

The expression of some circRNAs is up-regulated in gastric cancer (**Table 1**). For example, a study detected by RT-PCR technology found that the expression level of circPVT1 was significantly higher than that in the corresponding adjacent normal tissue as a proliferation factor and prognostic marker in gastric cancer tissue (47). circHIPK3 is also found to be up-regulated in gastric cancer tissues and cells, and the expression level of circHIPK3 is significantly correlated with the TNM stage of gastric cancer patients (48). Similar results are as follows, the expression of circ_0006282 in gastric cancer tissues is significantly higher than that in its adjacent non-cancerous tissues, and the high expression of circ_0006282 was associated with tumor size, lymph node metastasis and TNM stage (49). The expression of hsa_circ_0010882 is significantly up-regulated in gastric cancer patient plasma and gastric cancer cell lines, and its expression level is significantly correlated with the tumor size and histological grade of the patients (50). The expression level of hsa_circ_0000467 in gastric cancer tissues is significantly higher than that in the corresponding adjacent tissues, and it is correlated with the histological grade of gastric cancer (51). A study quantitatively detected the expression of circRBM33 in 79 pairs of GC tissues and paracancerous tissues and 4 GC cell lines (MGC-803, BGC-823, SGC-7901 and AGS) by RT-PCR. It was significantly up-regulated in GC tissue specimens and cell lines, and the expression level of circRBM33 is observed to be closely related to the clinical characteristics of GC patients (52). In the analysis of the expression levels of circURI1 in GC and adjacent normal tissues, and found that circURI1 is generally and significantly up-regulated in GC compared with adjacent tissues, and the decreased expression level of circURI1 was associated with TNM stage (III-IV) and distant metastasis (53). CircNHSL1 is the most up-regulated circRNA in gastric cancer metastatic tissues through RNA-seq analysis in 3 gastric cancer tissues with metastasis and 2 gastric cancer tissues without metastasis, and the high expression of circNHSL1 is positively correlated with UICC stage, pathological T stage, lymphatic metastasis, distant metastasis and histological grade. Meanwhile, the expression level of circNHSL1 in M1 stage tissues is higher than that in M0 stage tissues, and it is associated with progression and poor prognosis (54). In analyzing the expression of ebv-circLMP2A in 69 EBVa GC patients. It is found that high expression of ebv-circLMP2A is significantly associated with lymph node metastasis, distant metastasis and TNM advanced stage (55). The expression level of circDLST is significantly increased in gastric cancer tissues compared with adjacent tissues, and it is an independent prognostic factor for poor survival of gastric cancer patients. Among patients receiving chemotherapy (oxaliplatin plus 5-Fu), patients with high circDLST expression have shorter overall survival than those with low expression (56). Circ_ASAP2 is overexpressed in DDP-resistant gastric cancer tissues and cells, the down-regulation of circ_ASAP2 promote the sensitivity and apoptosis of DDP-resistant gastric cancer cells and inhibite cell proliferation, migration and invasion (57).

**Table 1:** Summary of some tumor-related CircRNAs.

CircRNA ID	Expression	Tumor size	Differentiation grade	T stage	TNM stage	Lymphatic metastasis	Distant metastasis	Drug resistance	Mechanisms
CircRNA ciRS-7	↑								CircRNA ciRS-7/MiR-7/PTEN/PI3K/AKT
Hsa_circ_0007507	↑			✔	✔	✔			Unknown
Hsa_circ_0110389	↑		✔	✔	✔	✔	✔		HsacircOl l0389/MiR-l27-5p or miR-l36-5p/SORT **l**
CircTMEM87A	↑	✔				✔			CircTMEM87A/MiR-l42-5p/ULKl
CircRNA UBE2Q2	↑	✔				✔			CircRNA UBE2Q2/MiR-370-3p/STAT3
CircDNA2	↑								CircDNA2/miR-l49-5p/CCDC6
CircLM07	↑			✔	✔				CircLMO7/MiR30a-3p/WNT2/|3-catenin
CircURIl	↑					✔	✔		CircUIl/hnRNPM
CircHIPK3	↑								CircHIPK3/miR-637/AKT **l**
Hsa_circ_0001020	↑						✔		Unknown
CircAG02	↑		✔		✔	✔			CircAG02/HURR
Circ-DONSON	↑				✔	✔			Circ-DONSON/NURF/SOX4
CircNRIPl	↑	✔				✔			CircNRIPl/MiRl49-5p/AKT l/mTOR
Circ-RanGAPl	↑	✔			✔	✔			Circ RanGAP1/MiR-877-3p/VEGFA
CircSHKBPl	↑	✔	✔		✔				CircSHKBP **l** *M* iR582-3p /HUR/VEGF/H SP90
CircNHSLl	↑		✔		✔	✔	✔		CircNHSLl/MiR-l3063p/SIXl/vimentin
CircAXINl	↑		✔		✔	✔			CircAXIN **l** /AXIN **l** -295aa/Wnt/|3-catenin
EBV-CircLMP2A	↑				✔	✔	✔		EBV-CircLMP2A/KHSRP/VHL/HIFla/VEGFA
Circ_SMAD4	↑								Circ_SMAD4/wnt/|3-catenin
CircHAS2	↑			✔	✔	✔			CircHAS2/MiR-944/PPM **I**E
CircRNA_l 00290	↑				✔	✔			CircRNA_l00290/MiR-29b-3p/lTGAl **l**
CircLMTK2	↑			✔	✔	✔			CircLMTK2/MiR-l505p/c-Myc
circ_0006282	↑	✔			✔	✔			Circ_0006282/MiR-l55/FBXO22
hsa_circ_0010882	↑	✔	✔						Hsa_circ_00l0882/PI3K/Akt/mTOR
circRBM33	↑								CircRBM33/M lR-149/1L-6
CircDLST	↑				✔			5-FU resistance	CircDLST/MiR-502-5p/NRAS/MEKl/ ERKl/2
CircRNA AKT 3	↑	✔	✔		✔			cisplatin resistance	CircRNA AKT3/MrR-l98/PIK3Rl
CircFAM73A	↑	✔			✔			cisplatin resistance	CircFAM73A/MiR-490-3p/HMGA2/hnrnpk/|3-cateniri
CircPVT 1	↑							cisplatin resistance	CircP V**I**1/M lR-152-3p/HDGF
Circ_ASAP2	↑							cisplatin resistance	Crrc_ASAP2/MiR-330-3p/NT5E
CircCPM	↑							5-FU resistance	Circular CPM/MiR-21-3p/PRKAA2
CircFNl	↑			✔	✔			cisplatin resistance	CrrcFNl/MiR-182-5p
CircOl 10805	↑							cisplatin resistance	Circ_0110805/MiR-299-3p/ENDOPDI
Circ_0026359	↑							cisplatin resistance	Crrc_0026359/MiR-1200/POLD4
Circ_0000260	↑							cisplatin resistance	Circ_0000260/M lR-129-5p MMP11
CircMCTP2	↓	✔		✔	✔			cisplatin resistance	CircMCTP2/MiR-99a-5p/MTMR3
Circul2	↓	✔	✔		✔	✔		cisplatin resistance	Crrcul2/MiR142-3p/ROCK2
CircRNA YAP1	↓	✔			✔			5-FU sensitive	CircRNA YAPl/MiR-367-5p/p27 Kip 1
Circ_0001017	↓							cisplatin resistance	Circ_0001017/MrR-543/PHLPP2
Hsa_circ_0009172	↓		✔		✔				Hsa_circ_0009172/MiR-485-3p/NTRK3
CircDIDOl	↓	✔					✔		CrrcDIDO 1/DIDO1-529aa/PRDX2
Circ0007360	↓								Crrc0007360/MiR-762/[RF7
CircGSK3B	↓	✔			✔	✔			CircGSK3B/EZH2/RORA
Circ-HuR	↓				✔	✔	✔		Crrc-HuR/CNBP/HuR
CircMRPS35	↓	✔			✔	✔			CircMRPS35/KAT7
CircCCDC9	↓	✔			✔	✔			CrrcCCDC9/MiR-67923p/CAVl
CircFATl(e2)	↓				✔	✔	✔		CircFATl(e2)/MiR-548gYBXl
CircRNA ST3GAL6	↓	✔			✔				CircRNA ST3GAL6/FOXP2/MET/mTOR
CircPTK2	↓					✔			CrrcPT K2/M rR-196a-3p /AAT K
CircMTOl	↓								CircM TOl/MiR-3200-5p /PEBP1
Hsa_crrc_0004872	↓	✔		✔		✔			Hsa_crrc_0004872/M rR-224/ Smad4/ADAR1
CircRNA_LARP4	↓								CircRNA_LARP4/MiR-424-5p/LAT S**I**
CircPSMC3	↓				✔	✔			CrrcPSM C3/M rR-296-5p
CircST3GAL6	↓	✔							CircST3GAL6/FOXP2/MET/mTOR
CircRAB31	↓	✔			✔	✔			CircRAB31/MiR-885-5p/PTEN/PI3K/AKT
Hsa_crrc_00001649	↓								Unknown
Hsa_circ_0003159	↓								Hsa_circ_0003159 / MiR-223-3p/NDRGl
Circ-KIAA1244	↓				✔	✔			Unknown
Circ-SMAD7	↓								Unknown

↑/↓, up-regulation and down-regulation; ✔, CircRNAs are associated with clinicopathological features.

### Down-regulated circRNAs in gastric cancer

Some circRNAs are down-regulated in gastric cancer. For example, circRNA LARP4 is lowly expressed in gastric cancer tissue, and its expression level is significantly correlated with the pathological stage and overall survival rate of gastric cancer patients (58). Hsa_circ_00001649 is significantly down-regulated in tumor tissue and serum of patients with gastric cancer, and its expression may be related to the type of gastric cancer, with relatively high sensitivity and specificity (59). hsa_circ_0003159 is lowly expressed in GC tissues and cells, and low expression of hsa_circ_0003159 is associated with lower overall and disease-free survival in gastric cancer patients (60). Similar results are as follows, a study showed that circ-KIAA1244 in GC tissues, plasma and cells was significantly lower compared with normal controls by analysing the circRNA expression profiles in plasma samples from 10 GC patients with different TNM stages and 5 healthy people, and further clinical data analysis showed that the low expression of circ-KIAA1244 in plasma was negatively correlated with TNM stage, lymph node metastasis and overall survival time in GC patients (61). Another study showed that circ-SMAD7 expression was significantly reduced in GC tissues compared with adjacent normal tissues (62). circYAP1 was significantly lower in gastric cancer tissues than in adjacent normal tissues. In addition, the treatment outcome of gastric cancer patients with high circYAP1 expression was better than those with low circYAP1 expression by observing 75 gastric cancer patients who received adjuvant chemotherapy (oxaliplatin and 5-Fu) (63). The expression level of circ_0001017 in GC tissues of recurrent patients was lower than that of primary patients by analysing 26 patients with primary gastric cancer (sensitive) and 33 patients with recurrent gastric cancer (resistant), and low expression of circ_0001017 was associated with CDDP resistance in GC (64).

### Relationship between circRNA and the occurrence and development of gastric cancer

Studies have found that some circRNAs may act as oncogenes to promote the occurrence and development of gastric cancer. For example, circ_0006282 can promote the proliferation and metastatic capacity of GC cells *in vitro* by acting as a miR-155 molecular sponge, and can promote tumor growth *in vivo* (49). The research results also showed that knockdown of hsa_circ_0010882 inhibited the proliferation, migration and invasion of gastric cancer cell lines, and increased apoptosis. In addition, the overexpression of hsa_circ_0010882 can enhance the proliferation, migration and invasion of gastric cancer cell, without changing apoptosis (50). Hsa_circ_0000467 promotes the proliferation and invasion ability of gastric cancer cells and the number of cells entering G2/M phase by regulating the expression of miR-326-3p (51). On the other hand, some circRNAs may act as tumor suppressor genes to inhibit the occurrence and development of gastric cancer. For example, circRBM33 inhibits gastric cancer cell apoptosis and promotes cell proliferation, migration and invasion through the circRBM33/miR-149/IL-6 axis (52). Hsa_circ_0003159 inhibits the proliferation, migration and invasion but induces apoptosis of GC cells by regulating miR-223-3p and NDRG1 (60). CircRHOBTB3 affects p21 protein expression by acting as a sponge for miR-654-3p, and then inhibits gastric cancer cell proliferation *in vitro* and xenograft tumor growth *in vivo* (65).

### circRNA as a diagnostic marker for gastric cancer

Although the current treatment technology for gastric cancer has been greatly improved, the overall survival of gastric cancer patients is still unsatisfactory. The main reason is that many patients do not undergo early surgical resection in time, and the gastric cancer is found at a later stage and miss the best opportunity for surgery, resulting in a greatly reduced treatment effect. Therefore, the early screening and diagnosis of gastric cancer has become the key to improve the prognosis of patients (66). Histopathology is currently the gold standard for the diagnosis of gastric cancer, but it requires invasive procedures to obtain gastric tissue samples. In recent years, gastroscopic examination has been widely used in clinical practice. It can visually check the tissue lesions of the stomach and biopsy suspected tissues. However, because it is an invasive examination, it may cause some physical discomfort to the examiner, and has contraindications, so the application is still limited. Tumor marker detection is also a commonly used method to assist the diagnosis of gastric cancer. Carcinoembryonic antigen (CEA), alpha-fetoprotein (AFP), carbohydrate antigen 19-9 (CA19-9) and carbohydrate antigen 72- 4 (CA72-4) and other substances are of great significance for the diagnosis of tumors, but due to the lack of specificity, they can only be used as auxiliary diagnosis. Based on the above analysis, in order to improve the current status of early diagnosis of gastric cancer, it is of great urgent to explore less or even non-invasive, high-sensitivity and specific detection methods.

The inherent closed-loop structure of circRNA makes its expression relatively stable in tissues and blood, which makes it more likely to be a diagnostic marker for tumors. For example, circRNAs are highly stable in mammalian cells, and one specific circRNA hsa_circ_002059 may be a potential novel and stable biomarker for the diagnosis of gastric cancer (67). The expression level of hsa_circ_0000190 is significantly reduced in gastric cancer tissue and plasma, and its sensitivity and specificity in diagnosing gastric cancer are significantly better than those of CEA and CA19-9 (68). Besides, the expression level of hsa_circ_0000745 in gastric cancer tissue is related to the degree of tumor differentiation, and its expression level in plasma was related to the stage of tumor lymph node metastasis. At the same time, the combination of its plasma expression level and CEA level may be an effective marker for the diagnosis of gastric cancer (69). Likewise, hsa_circ_0001020 is significantly up-regulated in gastric cancer patient plasma compared with normal plasma and is significantly lower in both the postoperative group and the healthy group than the preoperative group by analysing plasma samples from preoperative and postoperative gastric cancer patients and healthy volunteers, subsequently ROC curve is constructed to determine the potential screening value of hsa_circ_0001020 in plasma. The AUC, sensitivity, specificity are 0.738, 46.55% and 97.83%, respectively. When CEA combined with hsa_circ_0001020 are used as plasma biomarkers, their AUC, sensitivity and specificity are 0.852, 66.7%, and 91.3%, respectively (70). CircPSMC3 is significantly downregulated in gastric cancer and correlated with poor prognosis, and the down-regulation of circPSMC3 is negatively correlated with TNM stage and lymph node metastasis, meanwhile, the area under the ROC curve (AUC) of circPSMC3 in differentiating GC-MS from normal GC-MS was 0.9326, with a sensitivity of 85.85% and a specificity of 95.24% (71). In addition, Gastric cancer patients with high expression of circYAP1 had better therapeutic effect than those with low expression of circYAP1 by observing 75 gastric cancer patients who received adjuvant chemotherapy (oxaliplatin and 5-Fu) (63). Therefore, the circRNAs have important guiding significance for the early diagnosis of gastric cancer and chemotherapy of advanced gastric cancer.

### circRNAs as therapeutic targets for gastric cancer

With the deepening of the concept of precision medicine and the rapid development of molecular biology technology, molecular targeted therapy has been widely used in clinical anti-tumor work. Molecular targeted therapy, also known as “bio-missile”, is targeted at a certain protein molecule or gene fragment in the discovered tumor cells to specifically kill tumor cells, thereby inhibiting tumor growth and control tumor recurrence and metastasis (72). At present, the therapeutic targets of gastric cancer mainly include HER-2, EGFR, PI3K, mTOR and c-Met, etc., and targeted drugs targeting these receptors and kinases have achieved certain results in the clinical application of gastric cancer treatment (73). With the continuous development of circRNA research in recent years, its regulatory mechanism has been increasingly discovered in gastric cancer. Among them, circRNA function in a variety of cancer pathways by regulating the expression of some tumor-related genes, providing new potential molecular targets for targeted therapy of gastric cancer (74). For instance, circPVT1 exhibits frequent gene fragment amplification in gastric cancer, and can upregulate the expression of target gene E2F2 by acting as a molecular sponge of miR-125b, promoting the growth and proliferation of gastric cancer cells, and the stability of its expression makes it possible as a potential therapeutic target for gastric cancer (47). Synthetic anti-nuclease digested circRNA sponges have been reported to inhibit the proliferation of gastric cancer cells and reduce the activity of miR-21 against downstream targets, including the tumor protein DAXX. This finding suggests that synthetic circRNA sponges represent a simple, effective, and convenient therapeutic strategy to target miRNA loss-of-function *in vitro*, and is expected to gain potential therapeutic applications in human patients (75).

### circRNA as a prognostic marker for gastric cancer

Medical technology continues to advance, the incidence and mortality of gastric cancer have been decreasing year by year in our country, but the prognosis of patients with advanced gastric cancer still needs to be improved (76). At present, tumor recurrence and metastasis after surgical treatment are the main factors affecting the prognosis of gastric cancer patients. A study screened 4 circRNAs significantly associated with postoperative recurrence in patients with stage III gastric cancer by gene chip technology, namely circRNA_101308, circRNA_104423, circRNA_104916 and circRNA_100269. Based on the above four circRNAs, a new risk prediction model was constructed for the recurrence of stage III gastric cancer patients after radical resection, and the model was more accurate than the traditional TNM staging and could better reflect the prognosis of patients (77). Another study found that the survival rate of gastric cancer patients with high expression of hsa_circ_0081143 was significantly lower than that of gastric cancer patients with low expression, indicating that hsa_circ_0081143 might be a potential prognostic marker for gastric cancer (78). In addition, the aforementioned circRNA_LARP4 could also be used as an independent prognostic factor for gastric cancer patients, and the overall survival time of patients with high circRNA_LARP4 expression was significantly longer than that of patients with low expression (58). At the same time, circPVT1 molecule can also be considered as a prognostic indicator of gastric cancer, the survival rate of gastric cancer patients with high expression of circPVT1 and low expression of PVT1 is significantly higher compared with gastric cancer patients with low expression of circPVT1 and high expression of PVT1, this joint detection of circPVT1 and PVT1 has greater application value for the prognosis evaluation of gastric cancer patients (47).

### Advantages and disadvantages of circRNA as a diagnostic and prognostic biomarker

Through the whole transcriptome analysis of human peripheral blood, a large number of repeatable circRNAs were identified, laying the foundation for the study of the potential of circRNA as a tumor biomarker (39, 79). Compared with traditional biomarkers, circRNA has the following characteristics. Firstly, circRNA has a closed ring structure and can resist the degradation of linear mRNA decay mechanism, thus showing excellent stability and long half-life (80, 81). Secondly, circRNA is highly conserved and highly expressed, making it easier to detect. In addition, circRNA is widely present in various body fluids (blood, saliva, urine and gastric juice) (82–85), making detection more convenient. Thirdly, the expression pattern of circRNA is highly specific, including cell specificity, tissue specificity and developmental specificity (39). The expression patterns and their diversity in different cell and tissue types and developmental stages are highly recognizable (86, 87). In summary, circRNA is expected to become an ideal clinical biomarker and therapeutic target and has been proved in many diseases. However, although circular RNAs have good application prospects, the research of circRNAs in cancer is still in its infancy. Compared to traditional markers, circRNAs lack common standards for reporting and naming circRNAs, available cancer-related RNA sequence data sets, and new technologies for detecting circRNAs (88).

## Discussion

This review systematically summarizes the formation and regulatory mechanism of circRNA, its biological function and its relationship with gastric cancer. As a class of non-coding RNA molecules that are stably expressed in eukaryotes, circRNAs have rich biological functions. CircRNAs can interact with miRNAs and RBPs to regulate gene expression, and many nuclear-localized circRNAs can also regulate gene transcription. Although circRNAs have always been classified as non-coding RNAs, with in-depth research in recent years, circRNAs have been found to have coding potential, and its encoded polypeptides have important biological effects. These findings not only deepen our understanding of circRNAs, but also remind us that many unknown areas in circRNA research worth exploring.

Gastric cancer is a complex disease caused by multiple factors. Its exact pathogenesis has not been elucidated, and the treatment strategy still needs to be further improved. Given its closed loop structure, circRNA can exist stably in tissues and plasma, and the expression of circRNA has obvious tissue specificity. Therefore these characteristics make circRNA more potential to be an effective disease diagnosis, treatment and prognosis marker. Studies have found that many circRNAs are up-regulated or down-regulated in gastric cancer tissue or plasma, and these abnormally expressed circRNA molecules can regulate the expression of certain tumor-related proteins through different mechanisms, thus affecting the occurrence and development of gastric cancer. The expression level of some circRNAs may have a certain correlation with the clinicopathological indicators and survival time of gastric cancer patients. The above findings reveal that circRNAs function in the early diagnosis, lesion progression, treatment and prognosis of gastric cancer. Nevertheless, the action mechanism of circRNAs is still unclear, and some circRNAs with lower expression levels have not been discovered due to the limitations of detection methods in gastric cancer. In short, our current understanding of circRNAs is still at the primary level, and many experimental techniques and research strategies are still immature. Therefore, the transition from basic theory to clinical practice is worth studying and discussing. However, it is believed that more valuable circRNA molecules will be discovered through continuous innovation and exploration of scientific researchers, providing more effective molecular targets for the diagnosis, treatment and prognosis of gastric cancer.

## Author contributions

(I) Conception and design: QZ. (II) Administrative support: QZ. (III) Provision of study materials or patients: P’AD, PY, PL, YT. (IV) Collection and assembly of data: P’AD, PY. (V) Data analysis and interpretation: P’AD. (VI) Manuscript writing: All authors. (VII) Final approval of manuscript: All authors.

## Funding

This work was supported by the Cultivating Outstanding Talents Project of Hebei Provincial Government Fund (No.2019012); Hebei public health committee county-level public hospitals suitable health technology promotion and storage project (No.2019024); Hebei University Science and Technology Research Project (No.ZD2019139)

## Conflict of interest

The authors declare that the research was conducted in the absence of any commercial or financial relationships that could be construed as a potential conflict of interest.

## Publisher’s note

All claims expressed in this article are solely those of the authors and do not necessarily represent those of their affiliated organizations, or those of the publisher, the editors and the reviewers. Any product that may be evaluated in this article, or claim that may be made by its manufacturer, is not guaranteed or endorsed by the publisher.

## References

[1] Jeck WR Sorrentino JA Wang K Slevin MK Burd CE Liu J . Circular RNAs are abundant, conserved, and associated with ALU repeats. RNA (2013) 19(2):141–57. doi: 10.1261/rna.035667.112 PMC3543092 23249747

[2] Zhang Y Zhang XO Chen T Xiang JF Yin QF Xing YH . Circular intronic long noncoding RNAs. Mol Cell (2013) 51(6):792–806. doi: 10.1016/j.molcel.2013.08.017 24035497

[3] Li Z Huang C Bao C Chen L Lin M Wang X . Exon-intron circular RNAs regulate transcription in the nucleus. Nat Struct Mol Biol (2015) 22(3):256–64. doi: 10.1038/nsmb.2959 25664725

[4] Ebbesen KK Kjems J Hansen TB . Circular RNAs: Identification, biogenesis and function. Biochim Biophys Acta (2016) 1859:163–8. doi: 10.1016/j.bbagrm.2015.07.007 26171810

[5] Vicens Q Westhof E . Biogenesis of circular RNAs. Cell (2014) 159(1):13–4. doi: 10.1016/j.cell.2014.09.005 25259915

[6] Barrett SP Peter LW Salzman J . Circular RNA biogenesis can proceed through an exon-containing lariat precursor. Elife (2015) 4(6):e07540–80. doi: 10.7554/eLife.07540 PMC4479058 26057830

[7] Wang J Zhang Y Liu L Yang T Song J . Circular RNAs: New biomarkers of chemoresistance in cancer. Cancer Biol Med (2021) 18(2):421–36. doi: 10.20892/j.issn.2095-3941.2020.0312 PMC8185855 33738995

[8] Jeck WR Sharpless NE . Detecting and characterizing circularRNAs. Nat Biotechnol (2014) 32(5):453–61. doi: 10.1038/nbt.2890 PMC4121655 24811520

[9] Petkovic S Müller S . RNA Circularization strategies *in vivo* and *in vitro* . Nucleic Acids Res (2015) 43(4):2454–65. doi: 10.1093/nar/gkv045 PMC4344496 25662225

[10] Stoddard BL . Homing endonucleases from mobile group I introns: Discovery to genome engineering. Mobile DNA (2014) 5(1):7. doi: 10.1186/1759-8753-5-7 24589358 PMC3943268

[11] Costa M Walbott H Monachello D Westhof E Michel F . Crystal structures of a group II intron lariat primed for reverse splicing. Science (2016) 354(6316):aaf9258. doi: 10.1126/science.aaf9258 27934709

[12] Liang D Wilusz JE . Short intronic repeat sequences facilitate circular RNA production. Genes Dev (2014) 28:2233–47. doi: 10.1101/gad.251926.114 PMC4201285 25281217

[13] Zhang XO Wang HB Zhang Y Lu X Chen LL Yang L . Complementary sequence-mediated exon circularization. Cell (2014) 159:134–47. doi: 10.1016/j.cell.2014.09.001 25242744

[14] Conn SJ Pillman KA Toubia J Conn VM Salmanidis M Phillips CA . The RNA binding protein quaking regulates formation of circRNAs. Cell (2015) 160(6):1125–34. doi: 10.1016/j.cell.2015.02.014 25768908

[15] Li X Liu CX Xue W Zhang Y Jiang S Yin QF . Coordinated circRNA biogenesis and function with NF90/NF110 in viral infection. Mol Cell (2017) 67(2):214–27. doi: 10.1016/j.molcel.2017.05.023 28625552

[16] Ivanov A Memczak S Wyler E Torti F Porath HT Piechotta M . Analysis of intron sequences reveals hallmarks of circular RNA biogenesis in animals. Cell Rep (2015) 10(2):170–7. doi: 10.1016/j.celrep.2014.12.019 25558066

[17] Aktas T Avs ar Ilık I Maticzka D Bhardwaj V Pessoa Rodrigues C Mittler G . DHX9 suppresses RNA processing defects originating from the alu invasion of the human genome. Nature (2017) 544:115–9. doi: 10.1038/nature21715 28355180

[18] Chen LL . The expanding regulatory mechanisms and cellular functions of circular RNAs. Nat Rev Mol Cell Biol (2020) 21(8):475–90. doi: 10.1038/s41580-020-0243-y 32366901

[19] Ashwal-Fluss R Meyer M Pamudurti NR Ivanov A Bartok O Hanan M . circRNA biogenesis competes with pre-mRNA splicing. Mol Cell (2014) 56(1):55–66. doi: 10.1016/j.molcel.2014.08.019 25242144

[20] Zhang Y Xue W Li X Zhang J Chen S Zhang JL . The biogenesis of nascent circular RNAs. Cell Rep (2016) 15:611–24. doi: 10.1016/j.celrep.2016.03.058 27068474

[21] Liang D Tatomer DC Luo Z Wu H Yang L Chen LL . The output of protein- coding genes shifts to circular RNAs when the pre- mRNA processing machinery is limiting. Mol Cell (2017) 68:940–954.e943. doi: 10.1016/j.molcel.2017.10.034 29174924 PMC5728686

[22] Hansen TB Jensen TI Clausen BH Bramsen JB Finsen B Damgaard CK . Natural RNA circles function as efficient microRNA sponges. Nature (2013) 495:384–8. doi: 10.1038/nature11993 23446346

[23] Memczak S Jens M Elefsinioti A Torti F Krueger J Rybak A . Circular RNAs are a large class of animal RNAs with regulatory potency. Nature (2013) 495:333–8. doi: 10.1038/nature11928 23446348

[24] Zheng Q Bao C Guo W Li S Chen J Chen B . Circular RNA profiling reveals an abundant circHIPK3 that regulates cell growth by sponging multiple miRNAs. Nat Commun (2016) 7(1):11215. doi: 10.1038/ncomms11215 27050392 PMC4823868

[25] Guo JU Agarwal V Guo H Bartel DP . Expanded identification and characterization of mammalian circular RNAs. Genome Biol (2014) 15:409. doi: 10.1186/s13059-014-0409-z 25070500 PMC4165365

[26] Hansen TB Kjems J Damgaard CK . Circular RNA and miR-7 in cancer. Cancer Res (2013) 73:5609–12. doi: 10.1158/0008-5472.CAN-13-1568 24014594

[27] Han Z Zhang Y Sun Y Chen J Chang C Wang X . ERβ-mediated alteration of circATP2B1 and miR-204-3p signaling promotes invasion of clear cell renal cell carcinoma. Cancer Res (2018) 78(10):2550–63. doi: 10.1158/0008-5472.CAN-17-1575 29490945

[28] Wang KF Sun Y Tao W Fei X Chang CS . Androgen receptor (AR) promotes clear cell renal cell carcinoma (ccRCC) migration and invasion *via* altering the circHIAT1/miR-195-5p/29a-3p/29c-3p/CDC42 signals. Cancer Lett (2017) 394:1–12. doi: 10.1016/j.canlet.2016.12.036 28089832

[29] Zang J Lu D Xu A . The interaction of circRNAs and RNA binding proteins: An important part of circRNA maintenance and function. J Neurosci Res (2020) 98(1):87–97. doi: 10.1002/jnr.24356 30575990

[30] Du W Yang W Liu E Yang Z Dhaliwal P Yang BB . Foxo3 circular RNA retards cell cycle progression *via* forming ternary complexes with p21 and CDK2. Nucleic Acids Res (2016) 44:2846–58. doi: 10.1093/nar/gkw027 PMC4824104 26861625

[31] Du WW Fang L Yang W Wu N Awan FM Yang Z . Induction of tumor apoptosis through a circular RNA enhancing Foxo3 activity. Cell Death Differ (2017) 24(2):357–70. doi: 10.1038/cdd.2016.133 PMC5299715 27886165

[32] Abdelmohsen K Panda AC Munk R Grammatikakis I Dudekula DB De S . Identification of HuR target circular RNAs uncovers suppression of PABPN1 translation by CircPABPN1. RNA Biol (2017) 14:361–9. doi: 10.1080/15476286.2017.1279788 PMC5367248 28080204

[33] Conn VM Hugouvieux V Nayak A Conos SA Capovilla G Cildir G . A circRNA from SEPALLATA3 regulates splicing of its cognate mRNA through r-loop formation. Nat Plants (2017) 3:17053. doi: 10.1038/nplants.2017.53 28418376

[34] Chen CY Sarnow P . Initiation of protein synthesis by the eukaryotic translational apparatus on circular RNAs. Science (1995) 268:415–7. doi: 10.1126/science.7536344 7536344

[35] Perriman R Ares MJ . Circular mRNA can direct translation of extremely long repeating-sequence proteins *in vivo* . RNA (1998) 4:1047–54. doi: 10.1017/S135583829898061X PMC1369681 9740124

[36] Chen XP Han P Zhou T Guo X Song X Li Y . circRNADb: A comprehensive database for human circular RNAs with protein-coding annotations. Sci Rep (2016) 6:34985. doi: 10.1038/srep34985 27725737 PMC5057092

[37] Legnini I Di Timoteo G Rossi F Morlando M Briganti F Sthandier O . Circ-ZNF609 is a circular RNA that can be translated and functions in myogenesis. Mol Cell (2017) 66:22–37. doi: 10.1016/j.molcel.2017.02.017 28344082 PMC5387670

[38] Pamudurti NR Bartok O Jens M Ashwal-Fluss R Stottmeister C Ruhe L . Translation of CircRNAs. Mol Cell (2017) 66:9–21. doi: 10.1016/j.molcel.2017.02.021 28344080 PMC5387669

[39] Tao M Zheng M Xu Y Ma S Zhang W Ju S . CircRNAs and their regulatory roles in cancers. Mol Med (2021) 27(1):94. doi: 10.1186/s10020-021-00359-3 34445958 PMC8393742

[40] Gu Y Wang Y He L Zhang J Zhu X Liu N . Circular RNA circIPO11 drives self-renewal of liver cancer initiating cells *via* hedgehog signaling. Mol Cancer (2021) 20(1):132. doi: 10.1186/s12943-021-01435-2 34649567 PMC8515748

[41] Wu N Yuan Z Du KY Fang L Lyu J Zhang C . Translation of yes-associated protein (YAP) was antagonized by its circular RNA *via* suppressing the assembly of the translation initiation machinery. Cell Death Differ (2019) 26(12):2758–73. doi: 10.1038/s41418-019-0337-2 PMC7224378 31092884

[42] Huang Q Guo H Wang S Ma Y Chen H Li H . A novel circular RNA, circXPO1, promotes lung adenocarcinoma progression by interacting with IGF2BP1. Cell Death Dis (2020) 11(12):1031. doi: 10.1038/s41419-020-03237-8 33268793 PMC7710735

[43] Fan C Qu H Xiong F Tang Y Tang T Zhang L . CircARHGAP12 promotes nasopharyngeal carcinoma migration and invasion *via* ezrin-mediated cytoskeletal remodeling. Cancer Lett (2021) 496:41–56. doi: 10.1016/j.canlet.2020.09.006 32931883

[44] Yang Y Fan X Mao M Song X Wu P Zhang Y . Extensive translation of circular RNAs driven by N(6)-methyladenosine. Cell Res (2017) 27:626–41. doi: 10.1038/cr.2017.31 PMC5520850 28281539

[45] Hamashima C Goto R . Potential capacity of endoscopic screening for gastric cancer in Japan. Cancer Sci (2017) 108(1):101–7. doi: 10.1111/cas.13100 PMC5276833 27727490

[46] Hashad D Elbanna A Ibrahim A Khedr G . Evaluation of the role of circulating long non-coding RNA H19 as a promising novel biomarker in plasma of patients with gastric cancer. J Clin Lab Anal (2016) 30(6):1100–5. doi: 10.1002/jcla.21987 PMC6807052 27184562

[47] Chen J Li Y Zheng Q Bao C He J Chen B . Circular RNA profile identifies circPVT1 as a proliferative factor and prognostic marker in gastric cancer. Cancer Lett (2017) 388:208–19. doi: 10.1016/j.canlet.2016.12.006 27986464

[48] Wei J Xu H Wei W Wang Z Zhang Q De W . circHIPK3 promotes cell proliferation and migration of gastric cancer by sponging miR-107 and regulating BDNF expression. Onco Targets Ther (2020) 13:1613–24. doi: 10.2147/OTT.S226300 PMC7041611 32110057

[49] He Y Wang Y Liu L Liu S Liang L Chen Y . Circular RNA circ_0006282 contributes to the progression of gastric cancer by sponging miR-155 to upregulate the expression of FBXO22. Onco Targets Ther (2020) 13:1001–10. doi: 10.2147/OTT.S228216 PMC6999548 32099403

[50] Peng YK Pu K Su HX Zhang J Zheng Y Ji R . Circular RNA hsa_circ_0010882 promotes the progression of gastric cancer *via* regulation of the PI3K/Akt/mTOR signaling pathway. Eur Rev Med Pharmacol Sci (2020) 24(3):1142–51. doi: 10.26355/eurrev_202002_20165 32096170

[51] Mo WL Jiang JT Zhang L Lu QiC Li J Gu WD . Circular RNA hsa_circ_0000467 promotes the development of gastric cancer by competitively binding to MicroRNA miR-326-3p. BioMed Res Int (2020) 2020:4030826. doi: 10.1155/2020/4030826 32090087 PMC7026707

[52] Wang N Lu K Qu H Wang H Chen Y Shan T . CircRBM33 regulates IL-6 to promote gastric cancer progression through targeting miR-149. BioMed Pharmacother (2020) 125:109876. doi: 10.1016/j.biopha.2020.109876 32044717

[53] Wang X Li J Bian X Wu C Hua J Chang S . CircURI1 interacts with hnRNPM to inhibit metastasis by modulating alternative splicing in gastric cancer. Proc Natl Acad Sci USA (2021) 118(33):e2012881118. doi: 10.1073/pnas.2012881118 34385309 PMC8379983

[54] Zhu Z Rong Z Luo Z Yu Z Zhang J Qiu Z . Circular RNA circNHSL1 promotes gastric cancer progression through the miR-1306-3p/SIX1/vimentin axis. Mol Cancer. (2019) 18(1):126. doi: 10.1186/s12943-019-1054-7 31438963 PMC6704702

[55] Du Y Zhang JY Gong LP Feng ZY Wang D Pan YH . Hypoxia-induced ebv-circLMP2A promotes angiogenesis in EBV-associated gastric carcinoma through the KHSRP/VHL/HIF1α/VEGFA pathway. Cancer Lett (2022) 526:259–72. doi: 10.1016/j.canlet.2021.11.031 34863886

[56] Zhang J Hou L Liang R Chen X Zhang R Chen W . CircDLST promotes the tumorigenesis and metastasis of gastric cancer by sponging miR-502-5p and activating the NRAS/MEK1/ERK1/2 signaling. Mol Cancer (2019) 18(1):80. doi: 10.1186/s12943-019-1015-1 30953514 PMC6449953

[57] Sun Y Ma J Lin J Sun D Song P Shi L . Circular RNA circ_ASAP2 regulates drug sensitivity and functional behaviors of cisplatin-resistant gastric cancer cells by the miR-330-3p/NT5E axis. Anticancer Drugs (2021) 32(9):950–61. doi: 10.1097/CAD.0000000000001087 34016832

[58] Zhang J Liu H Hou L Wang G Zhang R Huang Y . Circular RNA_LARP4 inhibits cell proliferation and invasion of gastric cancer by sponging miR-424-5p and regulating LATS1 expression. Mol Cancer (2017) 16(1):151. doi: 10.1186/s12943-017-0719-3 28893265 PMC5594516

[59] Li WH Song YC Zhang H Zhou ZJ Xie X Zeng QN . Decreased expression of Hsa_circ_00001649 in gastric cancer and its clinical significance. Dis Markers (2017) 2017:4587698. doi: 10.1155/2017/4587698 28167847 PMC5266807

[60] Wang J Lv W Lin Z Wang X Bu J Su Y . Hsa_circ_0003159 inhibits gastric cancer progression by regulating miR-223-3p/NDRG1 axis. Cancer Cell Int (2020) 20:57. doi: 10.1186/s12935-020-1119-0 32099530 PMC7031989

[61] Tang W Fu K Sun H Rong D Wang H Cao H . CircRNA microarray profiling identifies a novel circulating biomarker for detection of gastric cancer. Mol Cancer (2018) 17(1):137. doi: 10.1186/s12943-018-0888-8 30236115 PMC6147053

[62] Zhang XY Xu YY Chen WY . Upregulation of circular SMAD7 inhibits tumorigenesis of gastric cancer by reversing epithelial-To-Mesenchymal transition. Eur Rev Med Pharmacol Sci (2020) 24(3):1152–7. doi: 10.26355/eurrev_202002_20166 32096163

[63] Liu H Liu Y Bian Z Zhang J Zhang R Chen X . Circular RNA YAP1 inhibits the proliferation and invasion of gastric cancer cells by regulating the miR-367-5p/p27 Kip1 axis. Mol Cancer. (2018) 17(1):151. doi: 10.1186/s12943-018-0902-1 30336780 PMC6193296

[64] Zhang J Zha W Qian C Ding A Mao Z . Circular RNA circ_0001017 sensitizes cisplatin-resistant gastric cancer cells to chemotherapy by the miR-543/PHLPP2 axis. Biochem Genet (2022) 60(2):558–75. doi: 10.1007/s10528-021-10110-6 34313883

[65] Deng G Mou T He J Lv D Liu H Yu J . Circular RNA circRHOBTB3 acts as a sponge for miR-654-3p inhibiting gastric cancer growth. J Exp Clin Cancer Res (2020) 39(1):1. doi: 10.1186/s13046-019-1487-2 31928527 PMC6956561

[66] Vidal AF Ribeiro-Dos-Santos AM Vinasco-Sandoval T Magalhães L Pinto P Anaissi AKM . The comprehensive expression analysis of circular RNAs in gastric cancer and its association with field cancerization. Sci Rep (2017) 7(1):14551. doi: 10.1038/s41598-017-15061-w 29109417 PMC5673933

[67] Li P Chen S Chen H Mo X Li T Shao Y . Using circular RNA as a novel type of biomarker in the screening of gastric cancer. Clin Chim Acta (2015) 444(4):132–6. doi: 10.1016/j.cca.2015.02.018 25689795

[68] Chen S Li T Zhao Q Xiao B Guo J . Using circular RNA hsa_circ_0000190 as a new biomarker in the diagnosis of gastric cancer. Clin Chim Acta (2017) 466:167–71. doi: 10.1016/j.cca.2017.01.025 28130019

[69] Huang M He YR Liang LC Huang Q Zhu ZQ . Circular RNA hsa_circ_0000745may serve as a diagnostic marker for gastric cancer. World J Gastroenterol (2017) 23(34):6330–8. doi: 10.3748/wjg.v23.i34.6330 PMC5603500 28974900

[70] Yan J Shao Y Lu H Ye Q Ye G Guo J . Hsa_circ_0001020 serves as a potential biomarker for gastric cancer screening and prognosis. Dig Dis Sci (2021) 67(8):3753–62. doi: 10.1007/s10620-021-07211-y 34424459

[71] Rong D Lu C Zhang B Fu K Zhao S Tang W . Correction to: CircPSMC3 suppresses the proliferation and metastasis of gastric cancer by acting as a competitive endogenous RNA through sponging miR-296-5p. Mol Cancer (2020) 19(1):140. doi: 10.1186/s12943-020-01252-z 32907590 PMC7487545

[72] Li T Guo H Zhao X . Gastric cancer cell proliferation and survival is enabled by a cyclophilin B/STAT3/miR-520d-5p signaling feedback loop. Cancer Res (2016) 77(5):1225. doi: 10.1186/s12943-020-01252-z 28011625

[73] Farran B Muller S Montenegro RC . Gastric cancer management: Kinases as a target therapy. Clin Exp Pharmacol Physiol (2017) 44(6):613–22. doi: 10.1111/1440-1681.12743 28271563

[74] Lee SY Oh SC . Changing strategies for target therapy in gastric cancer. World J Gastroenterol (2016) 22(3):1179–89. doi: 10.3748/wjg.v22.i3.1179 PMC4716029 26811656

[75] Liu X Abraham JM Cheng Y Wang Z Wang Z Zhang G . Synthetic circular RNA functions as a miR-21 sponge to suppress gastric carcinoma cell proliferation. Mol Ther Nucleic Acids (2018) 13:312–21. doi: 10.1016/j.omtn.2018.09.010 PMC6197335 30326427

[76] Chen W Zheng R Zhang S Zeng H Xia C Zuo T . Cancer incidence and mortality in China, 2013. Cancer Lett (2017) 401:63–71. doi: 10.1016/j.canlet.2017.04.024 28476483

[77] Zhang Y Li J Yu J Liu H Shen Z Ye G . Circular RNAs signature predicts the early recurrence of stage III gastric cancer after radical surgery. Oncotarget (2017) 8(14):22936–43. doi: 10.18632/oncotarget.15288 PMC5410275 28206972

[78] Xue M Li G Fang X Wang L Jin Y Zhou Q . Hsa_circ_0081143 promotes cisplatin resistance in gastric cancer by targeting miR-646/CDK6 pathway. Cancer Cell Int (2019) 19:25. doi: 10.1186/s12935-019-0737-x 30733646 PMC6359821

[79] Memczak S Papavasileiou P Peters O Rajewsky N . Identification and characterization of circular RNAs as a new class of putative biomarkers in human blood. PloS One (2015) 10(10):e0141214. doi: 10.1371/journal.pone.0141214 26485708 PMC4617279

[80] Cocquerelle C Mascrez B Hétuin D Bailleul B . Mis-splicing yields circular RNA molecules. FASEB J (1993) 7(1):155–60. doi: 10.1096/fasebj.7.1.7678559 7678559

[81] Suzuki H Zuo Y Wang J Zhang MQ Malhotra A Mayeda A . Characterization of RNase r-digested cellular RNA source that consists of lariat and circular RNAs from pre-mRNA splicing. Nucleic Acids Res (2006) 34(8):e63. doi: 10.1093/nar/gkl151 16682442 PMC1458517

[82] Bahn JH Zhang Q Li F Chan TM Lin X Kim Y . The landscape of microRNA, piwi-interacting RNA, and circular RNA in human saliva. Clin Chem (2015) 61(1):221–30. doi: 10.1373/clinchem.2014.230433 PMC4332885 25376581

[83] Zhao SY Wang J Ouyang SB Huang ZK Liao L . Salivary circular RNAs Hsa_Circ_0001874 and Hsa_Circ_0001971 as novel biomarkers for the diagnosis of oral squamous cell carcinoma. Cell Physiol Biochem (2018) 47(6):2511–21. doi: 10.1159/000491624 29991057

[84] Kölling M Haddad G Wegmann U Kistler A Bosakova A Seeger H . Circular RNAs in urine of kidney transplant patients with acute T cell-mediated allograft rejection. Clin Chem (2019) 65(10):1287–94. doi: 10.1373/clinchem.2019.305854 31371281

[85] Shao Y Li J Lu R Li T Yang Y Xiao B . Global circular RNA expression profile of human gastric cancer and its clinical significance. Cancer Med (2017) 6(6):1173–80. doi: 10.1002/cam4.1055 PMC5463069 28544609

[86] Xu T Wu J Han P Zhao Z Song X . Circular RNA expression profiles and features in human tissues: A study using RNA-seq data. BMC Genomics (2017) 18(Suppl 6):680. doi: 10.1186/s12864-017-4029-3 28984197 PMC5629547

[87] Zhou T Xie X Li M Shi J Zhou JJ Knox KS . Rat BodyMap transcriptomes reveal unique circular RNA features across tissue types and developmental stages. RNA. (2018) 24(11):1443–56. doi: 10.1261/rna.067132.118 PMC6191709 30093490

[88] Kristensen LS Hansen TB Venø MT Kjems J . Circular RNAs in cancer: Opportunities and challenges in the field. Oncogene. (2018) 37(5):555–65. doi: 10.1038/onc.2017.361 PMC5799710 28991235

